# Risk factors for early–onset seizures after stroke: A systematicreview and meta‐analysis of 18 observational studies

**DOI:** 10.1002/brb3.2142

**Published:** 2021-05-04

**Authors:** Sitian Ma, Xiaoxuan Fan, Xiaoping Zhao, Kai Wang, Huan Wang, Yongfeng Yang

**Affiliations:** ^1^ Shaanxi University of Chinese Medicine Shaanxi China; ^2^ Affiliated Hospital of Shaanxi University of Chinese Medicine Shaanxi China

**Keywords:** case‐control study, early‐onset seizures, meta‐analysis, risk factors, stroke

## Abstract

**Objectives:**

To systematically evaluate the risk factors of early‐onset seizures after stroke, in order to better provide evidence‐based results for early detection, identification, targeted prevention, and treatment of this disease.

**Methods:**

PubMed, EMBASE, The Cochrane Library, CNKI, and WanFang databases were searched to collect relevant studies on the risk factors of early‐onset seizures after stroke from January 2010 to January 2020. Meta‐analysis of all included studies was performed by using RevMan version 5.3 and Stata version 14.0 software.

**Results:**

Eighteen case–control studies with a total sample size of 13,289 cases, including 813 cases with early‐onset seizures after stroke, and 12,476 cases with non‐early‐onset seizures after stroke were included. The results of meta‐analysis showed that cortical involvement [Odds Ratio (OR) = 5.00, 95%Confidence Interval (CI) (2.85, 8.74), *p* < .00001], cerebral infarction with hemorrhagic transformation [OR = 2.77, 95%CI (1.87, 4.11), *p* < .00001] and intracerebral hemorrhage [OR = 1.83, 95%CI (1.13, 2.97), *p* = .01]‐related factors showed greater association with the occurrence of early‐onset seizures after stroke.

**Conclusions:**

These findings suggest that cortical involvement, intracerebral hemorrhage, and cerebral infarction with hemorrhagic transformation are important predictors and risk factors for early seizures after stroke, while the patient's gender, age, NHISS score, alcoholism, smoking, high blood pressure, diabetes, atrial fibrillation, dyslipidemia, receiving surgical treatment, and reperfusion therapy showed no association with the occurrence of early‐onset seizures after stroke.

## Graphical abstract

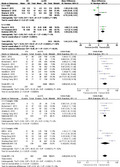

## 1 INTRODUCTION

Early‐onset seizures after stroke (ES) is defined as seizures that occur within 7 days after stroke onset (Fisher et al., 2014) and is regarded as a common complication after stroke. Biffi et al. (2016) have pointed out that about 10% of patients included in the study have diagnosed with early seizures within 7 days of intracerebral hemorrhage. Beghi et al. (2011) conducted a large multicenter study and found that the incidence of acute symptomatic seizures was 6.3% in patients with ischemic or hemorrhagic stroke. Van Tuijl JH and other researchers (Tuijl et al., 2018) have revealed that the disability and mortality rates in ES patients were significantly higher than those of non‐epileptic patients. The occurrence or recurrence of seizures symptoms in stroke patients may lead to unfavorable functional prognosis (Bentes et al., 2017; Huang et al., 2014), poor quality of life (Zelano, 2020), and higher mortality rate (Mohamed & Kissani, 2015). This negative effect not only exists in elderly stroke patients but also in young patients (Arntz et al., 2015).

At present, there are many studies (Abraira et al., 2020; Bian, 2017; Shehta et al., 2018) that discussed the risk factors for ES after stroke. The risk factors for individuals with ES included baseline characteristic factors, lifestyle‐related factors, underlying diseases‐related factors, brain injury‐related factors, and whether to receive surgery. For example, Abraira et al. (2020) have considered cerebral hemorrhage and cortical hemorrhage as risk factors for ES. Shehta et al. (2018) have believed that intracerebral hemorrhage, cortical lesions, and large lesion size as risk factors for ES. And Bian (2017) has believed that infarct lesions of 2–5 cm diameter and cortical lesions are related to the occurrence of ES. However, due to heterogeneity and diversity of the disease itself, the results of each study are different and occasionally contradictory. For example, Szaflarski et al. (2008) found that younger patients have a higher incidence of ES. However, researchers such as Procaccianti et al. (2012) and Wang et al. (2013) hold different views and believe that age is not a risk factor for ES. In terms of stroke severity factors, although Mohamed and Kissani (2015) and Goswami et al. (2012) found that the severity of stroke can be used as a risk factor for ES, especially Procaccianti et al. (2012) believes that ES may be considered a marker of stroke severity; Wang et al. (2013) believes that stroke patients are not associated with ES symptoms. The factors related to stroke subtypes are more controversial. Some studies (Abraira et al., 2020; Bladin et al., 2000; Goswami et al., 2012) have reported that hemorrhagic stroke is an independent risk factor for ES, Hundozi et al. (2016) has found that hemorrhagic and ischemic stroke patients have the same incidence of ES after stroke, while Aiwansoba and Chukwuyem (2014) has found that cerebral infarction is more related to ES. Therefore, this meta‐analysis was conducted on the risk factors of ES that are controversial and systematically evaluates the main risk factors that occur in order to provide better decision recommendations for guiding clinicians in early identification, prevention, diagnosis, and treatment.

## 2 MATERIALS AND METHODS

### 2.1 Literature retrieval strategy

The current meta‐analysis was conducted based on the Preferred Reporting Items for Systematic Reviews and Meta‐Analyses statement (PRISMA) (Reference). PubMed, EMBASE, the Cochrane Library, CNKI, and WANFANG databases were searched to identify relevant studies. The search was conducted from January 2010 to January 2020 using the search terms “after stroke”, “cerebral hemorrhage”, “brain ischemia”, “early seizures”, “risk factors”, and “factors”. The search words and keywords were also joined for conducting the search. The reference lists of eligible publications were manually checked to identify any other potential studies.

### 2.2 Literature inclusion and exclusion criteria

#### 2.2.1 Inclusion criteria

1. Each database (between 2010.01 and 2020.01) publishes one or more of the risk factors for early‐onset seizures after stroke in Chinese and English, such as gender, age, NIHSS score at admission, alcoholism, smoking, hypertension, diabetes, atrial fibrillation, dyslipidemia, hemorrhagic transformation, cortical injury, stroke subtype, receiving surgical treatment, etc.
2. Research subjects: studies that included patients with early‐onset seizures after stroke (ES group) and those with non‐early‐onset seizures after stroke (no‐ES group).
3. Diagnostic criteria: International League Against Epilepsy for ES is defined as the appearance of seizures symptoms within 7 days after a stroke, and the criteria for appearance of seizures symptoms within 14 days (2 weeks) after a stroke are also adopted by the researchers (Denier et al., 2010; Hundozi et al., 2016; Menon & Shorvon, 2009; Wang et al., 2013). Therefore, the ES diagnostic criteria used in this study are seizures within 14 days (2 weeks) after stroke, considering that as much sample size as possible should be taken.
4. Type of study: case–control studies.
5. Statistical data: studies with clear original data or odd's ratio OR [(95% confidence interval (CI)], or the above data can be calculated.
6. In case of publication of multiple articles by the authors from the same institution using overlapping sample data, only recent studies or studies with more complete information are selected.

#### 2.2.2 Exclusion criteria

1. Summary report, meeting abstracts, commentaries, republished articles, etc.
2. Literature with no clear sample source, no control group, or inconsistent control group definition.
3. Studies on children.
4. Studies with Newcastle‐Ottawa Scale (NOS) score of ≤4.

### 2.3 Document extraction

The literature was screened according to the inclusion and exclusion criteria, and the following information was extracted: the first author's name, publication time, nationality of study population, total number of samples in case and control groups, average age, possible risk factors, details of exposure factors, etc.

### 2.4 Study quality of risk of bias

Two investigators (MA Sitian and WANG Huan) have independently evaluated the risk of bias of included studies. If there are inconsistent opinions, then a third author (YANG Yongfeng) was contacted for decision. Third‐party opinions will be looked out if still differences exist. The bias risk evaluation of case–control studies was done using the NOS scale in order to evaluate the quality of literature included, in which a score of 9 points, ≥ 7 points were considered as high‐quality, 4 to 6 points were considered as medium‐quality articles, and ≤3 points were considered as low‐quality articles (Table 2).

### 2.5 Statistical methods

Statistical analysis was performed using Review Manager version 5.3 software. *I* ^2^ was used to evaluate heterogeneity. When *p* > .1 and *I* ^2^ < 50%, a fixed‐effects model is used. Otherwise, a random‐effects model was used. A subgroup analysis was performed based on factors such as sample size, country, and region to find the source of heterogeneity. In case–control studies, the odds ratios (OR) was used as the effect scale and its 95% confidence intervals (CI) was calculated at the same time, with *p* ≤ .05 as statistically significant difference. The man differences (MD) were used as the effect scale for continuous variables, and its 95% CI was calculated at the same time. The difference was statistically significant with *p* ≤ .05. Sensitivity analysis was used to calculate combined OR value and 95% CI, and compared the two sets of results to show whether the results are stable. Egger funnel chart was drawn using Stata version 14.0 software, and publication bias was evaluated by Egger *p* value result and whether the funnel chart was symmetrical or not. If *p* > .10 and funnel chart showed no obvious asymmetry, this indicated no obvious publication bias. Otherwise, publication bias was indicated.

## 3 RESULTS

### 3.1 Literature search results

**FIGURE 1:**
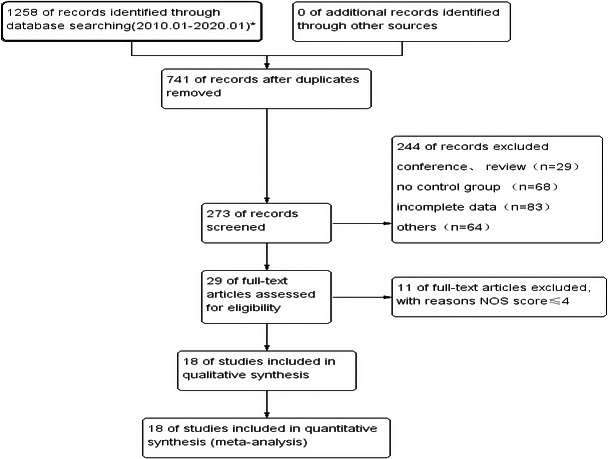
Literature search process and results. * The searched databases and detected documents are as follows: PubMed (*n* = 445), EMbase (*n* = 217), The Cochrane Library (*n* = 19), CNKI (*n* = 232), WanFang data (*n* = 345)

A total of 1,258 documents were initially retrieved, and after the elimination research layer by layer, 18 studies were finally included (Abraira et al., 2020; Aiwansoba & Chukwuyem, 2014; Arntz et al., 2013; Bian, 2017; Biffi et al., 2016; Chen & Du, 2018; Denier et al., 2010; Gao, 2016; Goswami et al., 2012; Hundozi et al., 2016; Mohamed & Kissani, 2015; Pezzini et al., 2013; Procaccianti et al., 2012; Serafini et al., 2015; Shehta et al., 2018; Wang et al., 2013; Yang, 2012; Zeng, 2018), including a total of 13,289 research objects. (Figure 1).

### 3.2 Baseline characteristics of research and assessment of bias risk

A total of 18 case–control studies were included, and 6 (Bian, 2017; Chen & Du, 2018; Gao, 2016; Wang et al., 2013; Yang, 2012; Zeng, 2018) studies were conducted in Chinese population, 3 (Pezzini et al., 2013; Procaccianti et al., 2012; Serafini et al., 2015) studies were conducted in Italy, and the USA (Biffi et al., 2016), the Netherlands (Arntz et al., 2013), Spain (Abraira et al., 2020), France (Denier et al., 2010), Morocco (Mohamed & Kissani, 2015), India (Goswami et al., 2012), Egypt (Shehta et al., 2018), Kosovo (Hundozi et al., 2016), and Nigeria (Aiwansoba & Chukwuyem, 2014) each have one study (Table 1). The NOS scale was used to conduct bias risk assessment to evaluate the quality of 18 included studies, wherein three studies (Biffi et al., 2016; Pezzini et al., 2013; Serafini et al., 2015) had a score of 8 points, 5 (Abraira et al., 2020; Arntz et al., 2013; Procaccianti et al., 2012; Shehta et al., 2018; Wang et al., 2013) had 7 points, 5 (Aiwansoba & Chukwuyem, 2014; Bian, 2017; Denier et al., 2010; Goswami et al., 2012; Hundozi et al., 2016) had 6 points, and 5 (Chen & Du, 2018; Gao, 2016; Mohamed & Kissani, 2015; Yang, 2012; Zeng, 2018) had 5 points. All studies were of medium‐to‐high‐quality, showing no bias risk of case–control studies (Table 2).

**TABLE 1:** The characteristics of the included studies

Study	Country / region	Number of cases		Age (Mean ± *SD*)	Control source	Risk factors
		Case group (ES)	Control group (no‐ES)			
Abraira et al. (2020)	Spain	38	926	72.3 ± 13.2	Hospital	a,b,d,e,f,g,h,i,j,l,n
Shehta et al. (2018)	Egypt	14	136	60.8 ± 12.5	Multi‐center	a,b,c,e,f,g,i,j,k,l
Hundozi et al. (2016)	Kosovo	39	903	69 ± 12	Hospital	j,n
Biffi et al.(2016)	United States	86	786	71.0 ± 12.3	Community	a,f,k
Serafini et al. (2015)	Italy	39	743	80 (71–86)	Community	a,e,f,g,h,i,j,k
Denier et al. (2010)	France	14	314	63.9 ± 14.9	Hospital	a,b,c,e,f,g,j2
Mohamed & Kissani (2015)	Morocco	47	305	71.6 ± 14.6	Hospital	b,c,e,f,g,h,i,l,k
Aiwansoba & Chukwuyem (2014)	Nigeria	25	226	59.97 ± 13.32	Hospital	a,b,j
Arntz et al. (2013)	Netherlands	25	672	40.5 ± 7.8	Hospital	a,j
Pezzini et al. (2013)	Italy	20	496	65.5 ± 15.2	Both	c,j,k,l
Procaccianti et al. (2012)	Italy	66	1 987	82 ± NG	Hospital	a,f,g,h,l
Goswami et al. (2012)	India	79	362	72.3 ± 13.2	Hospital	a,b,c,d,e,f,g,i,j,k
Wang et al. (2013)	China	123	1 862	57.2 ± NG	Hospital	j
Zeng (2018)	China	63	497	58.7 ± 6.6	Hospital	a,d,e,g,k
Chen & Du (2018)	China	12	64	66.3 ± NG	Hospital	k
Bian (2017)	China	31	987	NG	Hospital	a,b,d,e,f,g,k,j,m
Gao (2016)	China	21	1,056	73 ± NG	Hospital	a,e,f,g,i,l
Yang (2012)	China	71	104	68.72 ± 10.45	Hospital	a,b,k,m

Note

a. Gender;b. Age;c. NIHSS at admission; d. Alcoholism; e. Smoking; f. Hypertension; g. Diabetes; h. Atrial fibrillation; i. Dyslipidemia; j. Stroke subtype (j1: cerebral hemorrhage, j2: cerebral infarction); k. Cortex Injury (cortical hemorrhage); l. Cerebral infarction with hemorrhagic transformation; m. Surgical treatment; n. Reperfusion therapy;ES: Early‐onset seizures after stroke; NG: The original text did not provide information;Both:Community and hospital.

**TABLE 2:** Results of bias risk assessment included in case‐control studies

Study	Selection				Comparability	Exposure			Score
	adequate definition of case	Representativeness of the cases	Selection of Controls	Definition of Controls		Ascertainment of exposure	Same method of ascertainment for cases and controls	Non‐ Response rate	
Abraira et al. (2020)	★	★		★	★★	★	★		7
Shehta et al. (2018)	★	★	★	★	★		★	★	7
Hundozi et al. (2016)	★	★		★	★		★	★	6
Biffi et al.(2016)	★	★	★	★	★	★	★	★	8
Serafini et al. (2015)	★	★	★	★	★	★	★	★	8
Denier et al. (2010)	★	★		★		★	★	★	6
Mohamed & Kissani (2015)	★	★		★			★	★	5
Aiwansoba & Chukwuyem (2014)	★	★		★		★	★	★	6
Arntz et al. (2013)	★	★		★	★	★	★	★	7
Pezzini et al. (2013)	★	★	★	★	★	★	★	★	8
Procaccianti et al. (2012)	★	★		★	★	★	★	★	7
Goswami et al. (2012)	★	★		★	★		★	★	6
Wang et al. (2013)	★	★		★	★	★	★	★	7
Zeng (2018)	★	★		★	★		★		5
Chen & Du (2018)	★	★		★			★	★	5
Bian (2017)	★	★		★	★	★	★		6
Gao (2016)	★	★		★	★		★		5
Yang (2012)	★	★		★			★	★	5

Note

In the “Selection” and “Exposure” categories, a quality item of a study can be rated at most one “★”, and for the “Comparability” category, at most two “★”.

### 3.3 Results of meta‐analysis of major risk factors

#### 3.3.1 Factors related to baseline characteristic

A meta‐analysis of baseline characteristic factors such as the patient's gender, age, NHISS score, and ES occurrence (Table 3) has been carried out. The results show that there is no heterogeneity in the characteristic factors of gender and age (*I* ^2^ = 0, *p* > .10), so the fixed effects model was adopted; the characteristic factors of NIHSS at admission were heterogeneous (*I* ^2^ = 92%, *p* < .000 01), so the random effects model was adopted. Meta‐analysis results show that (Table 3): the above‐mentioned baseline characteristic factors are not statistically significant in association with ES, suggesting that gender, age, and NIHSS at admission are not risk factors for ES.

**TABLE 3:** Meta‐analysis results: baseline characteristics, lifestyle habits and basic diseases

Risk factors	Number of documents involved	Total sample size	Heterogeneity test			Effect model	OR (95%Cl)	*p*
			*Q*	*p*	*I* ^2^ (%)			
Factors related to baseline characteristics								
Gender	8	9,368	10.06	0.61	0	FE	1.00 (0.84,1.20)	.97
Age*	13	3,679	5.86	0.56	0	FE	−0.54 (−2.07,0.99)	.49
NIHSS at admission*	5	1787	50.37	<0.00001	92	RE	1.44 (−2.21,5.08)	.44
Factors related to lifestyle habits								
Alcoholism	4	2,933	37.39	<0.000 01	92	RE	2.46 (0.46,13.14)	.29
Smoking	9	1617	8.11	0.42	1	FE	1.02 (0.80,1.31)	.87
Related factors of basic diseases								
Hypertension	10	8,037	0.96	1.00	0	FE	0.91 (0.74,1.12)	.38
Diabetes	10	7,725	19.89	0.02	55	RE	1.18 (0.82,1.69)	.38
Atrial fibrillation	4	4,151	6.66	0.08	55	RE	0.78 (0.46,1.34)	.37
Dyslipidemia	6	3,766	1.34	0.93	0	FE	0.92 (0.67,1.26)	.59

Note

The data effect size of the risk factors marked with “*” is MD (95% Cl).

**FIGURE 2:**
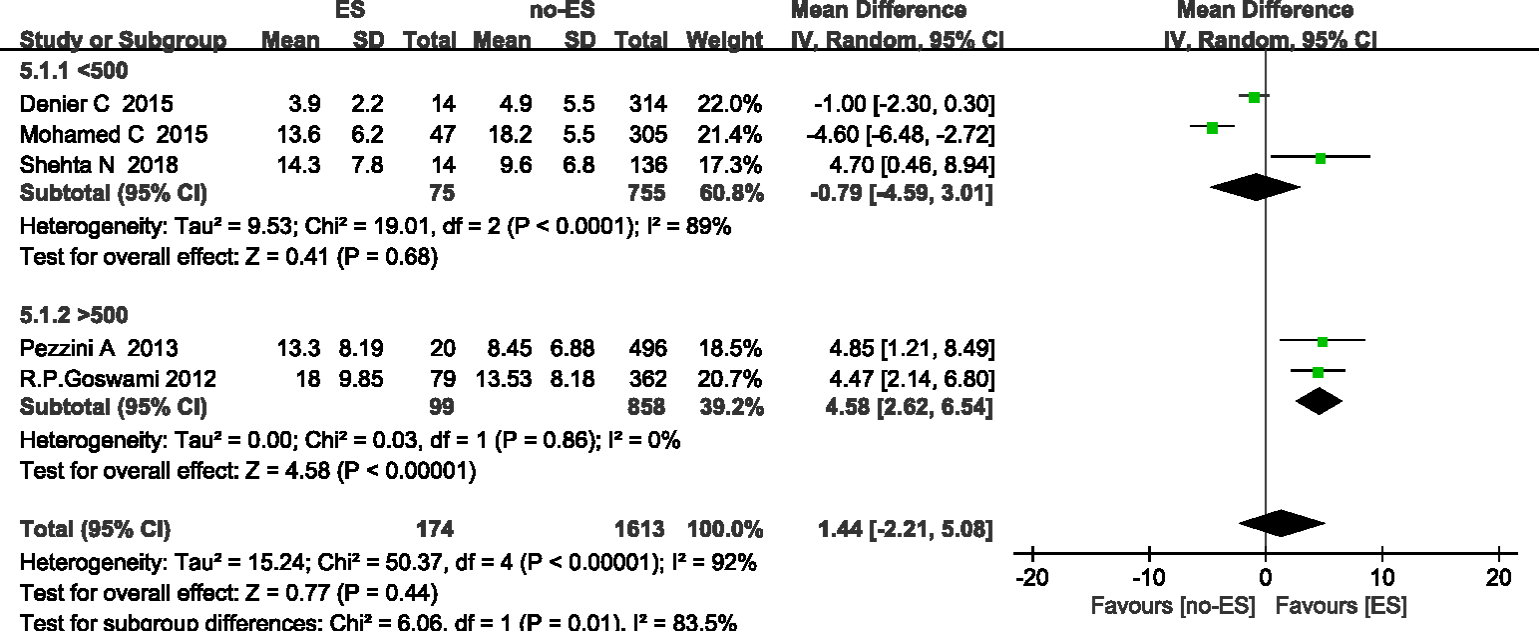
NIHSS at admission and ES: Subgroup analysis based on total sample size

According to the difference of the sample size, a subgroup analysis about the characteristic factor of NIHSS at admission has been carried out (Figure 2) and the results showed that: when the sample size is >500, the analysis result is statistically significant [MD (95%Cl) = 4.58 (2.62, 6.53), *p* < .000 01]. Therefore, it can be determined that when the sample size is greater than 500, the degree of neurological deficit is correlated with the occurrence of ES.

#### 3.3.2 Factors related to lifestyle habits

Four studies (Abraira et al., 2020; Bian, 2017; Goswami et al., 2012; Zeng, 2018) evaluated the relationship between alcoholism and ES (Table 3). A total of 2,933 samples were included, of which there were 161 cases in the ES group after stroke, and 2,772 cases in the no‐ES group after stroke. After extracting the data and calculating it, *I* ^2^ = 92%, *p* < .000 01, suggested the existence of heterogeneity. Therefore, random effect model was used, and the results showed no statistical significance [OR (95%Cl) = 2.46 (0.46, 13.14), *p* = .29]. Research suggests that there is significant heterogeneity in alcoholism factors, so subgroup analysis is carried out according to sample size and different countries (Table 4 and Table 5), in which the results showed no changes and had no effect on the outcome indicators. Nine articles (Abraira et al., 2020; Bian, 2017; Denier et al., 2010; Gao, 2016; Goswami et al., 2012; Mohamed & Kissani, 2015; Serafini et al., 2015; Shehta et al., 2018; Zeng, 2018) reported the relationship between smoking and ES (Table 3). A total of 1617 samples were included. Among these, there were 117 samples in the ES group after stroke, and 1559 in the no‐ES group after stroke. A fixed‐effects model (*I* ^2^ = 1%, *p* = .42) was used, and the results showed no statistical significance [OR (95% Cl) = 1.02 (0.80, 1.31), *p* = .87].

**TABLE 4:** Lifestyle habits and basic diseases: Subgroup analysis based on total sample size

Factors	Sample size	Number of documents	Heterogeneity test			OR (95%Cl)	*p*
			*Q*	*p*	*I* ^2^ (%)		
Alcoholism	<500	2 (Goswami et al., 2012; Zeng, 2018)	29.45	<0.000 01	97	9.10 (0.13, 628.26)	.31
	>500	2 (Abraira et al., 2020; Bian, 2017)	2.13	0.14	53	0.71 (0.24, 2.11)	.53
Diabetes	<500	5 (Denier et al., 2010; Goswami et al., 2012; Mohamed & Kissani, 2015; Shehta et al., 2018; Zeng, 2018)	8.14	0.09	51	1.68 (1.04, 2.70)	.03
	>500	5 (Abraira et al., 2020; Bian, 2017; Gao et al., 2016; Procaccianti et al., 2012; Serafini et al., 2015)	1.14	0.89	0	0.79 (0.56, 1.13)	.22
Atrial fibrillation	<500	1 (Mohamed & Kissani, 2015)	–	–	–	0.52 (0.22, 1.20)	–
	>500	3 (Abraira et al., 2020; Biffi et al., 2016; Procaccianti et al., 2012)	5.07	0.08	61	0.87 (0.46, 1.64)	.67

**TABLE 5:** Lifestyle habits and basic diseases: Subgroup analysis of Chinese and foreign population

Factors	Country	Number of documents	Heterogeneity test			OR (95%Cl)	*p*
			*Q*	*p*	*I* ^2^ (%)		
Alcoholism	China	2 (Bian, 2017; Zeng, 2018)	3.58	0.06	72	0.77 (0.29, 2.07)	.61
	Foreign	2 (Abraira et al., 2020; Goswami et al., 2012)	18.26	<0.000 1	95	9.92 (0.17, 574.20)	.27
Diabetes	China	3 (Bian, 2017; Gao et al., 2016; Zeng, 2018)	9.13	0.01	78	1.36 (0.49, 3.77)	.56
	Foreign	7 (Abraira et al., 2020; Denier et al., 2010; Goswami et al., 2012; Mohamed & Kissani, 2015; Procaccianti et al., 2012; Serafini et al., 2015; Shehta et al., 2018)	7.41	0.28	19	1.07 (0.78, 1.47)	.66

#### 3.3.3 Related factors of basic diseases

Meta‐analysis on the relationship between the underlying disease factors related to hypertension, diabetes, atrial fibrillation, and dyslipidemia and the occurrence of ES (Table 3) was conducted. The results showed that there were no heterogeneity in the two factors of hypertension and dyslipidemia (*I* ^2^ = 0, *p* > .10), and the fixed‐effects model was adopted. While the two factors of diabetes and atrial fibrillation are heterogeneous (*I* ^2 ^= 55%, *p* < .10), and the random‐effects model was adopted. The results of meta‐analysis showed no statistically significant association between the above factors and ES, suggesting hypertension, diabetes, atrial fibrillation, and dyslipidemia as not risk factors for ES.

The study found significant heterogeneity in the factors of diabetes and atrial fibrillation, so we further conducted a subgroup analysis of different sample sizes and countries (Table 4 and Table 5). This can partially explain the source of heterogeneity related to diabetes, but the results of atrial fibrillation still remain unchanged.

#### 3.3.4 Factors related to the brain

##### Cortical injury

**FIGURE 3:**
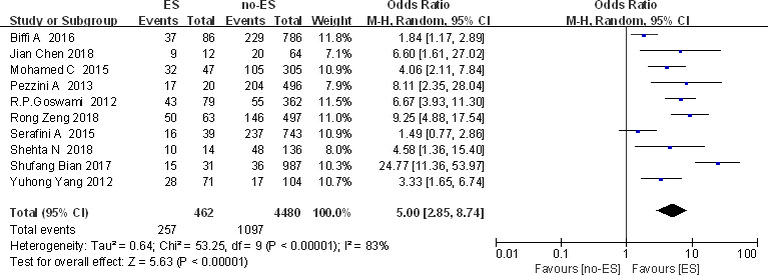
Forest plot: cortical injury and ES

Ten (Bian, 2017; Biffi et al., 2016; Chen & Du, 2018; Goswami et al., 2012; Mohamed & Kissani, 2015; Pezzini et al., 2013; Serafini et al., 2015; Shehta et al., 2018; Yang, 2012; Zeng, 2018) studies conducted a meta‐analysis of cortical injury and ES occurrence (Figure 3). A total of 462 cases were included in the ES group, in which 257 cases of ES patients were with cortical injury. A total of 4,480 cases were included in the no‐ES group, and 1,097 cases of no‐ES patients were with cortical injury. Heterogeneity (*I* ^2 ^= 83%, *p* < .000 01) was observed, and random‐effects model was used. The results showed that the composition ratio of cortical damage in ES group was greater than that in the no‐ES group [OR (95% Cl) = 5.00 (2.85, 8.74)]. The difference was statistically significant (*p* < .01), indicating cortical injury as a risk factor for ES after stroke.

**FIGURE 4:**
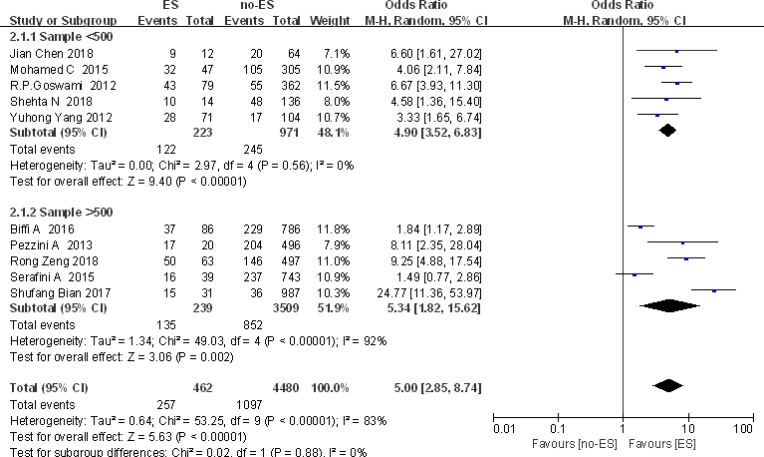
Cortical injury and ES: Subgroup analysis based on total sample size

The study found that there is significant heterogeneity in cortical damage factors, so we further conducted subgroup analysis according to different sample sizes (Figure 4) and regions (Asia/Europe/Africa). The results showed that the sample size can partially explain the source of heterogeneity of cortical damage factors. However, the results of subgroup analysis in different regions were unchanged.

##### Hemorrhagic transformation

**FIGURE 5:**
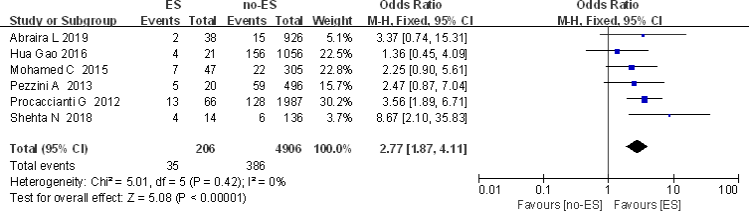
Forest plot: hemorrhagic transformation and ES

Six (Abraira et al., 2020; Gao, 2016; Mohamed & Kissani, 2015; Pezzini et al., 2013; Procaccianti et al., 2012; Shehta et al., 2018) studies reported hemorrhagic transformation and occurrence of ES (Figure 5). The data suggested that no heterogeneity (*I* ^2^ = 0), and fixed‐effects model was used. The results showed that OR (95% Cl) = 2.77 (1.87, 4.11), suggesting statistical significance and indicating that hemorrhagic transformation is a risk factor for ES.

##### Stroke subtype

**FIGURE 6:**
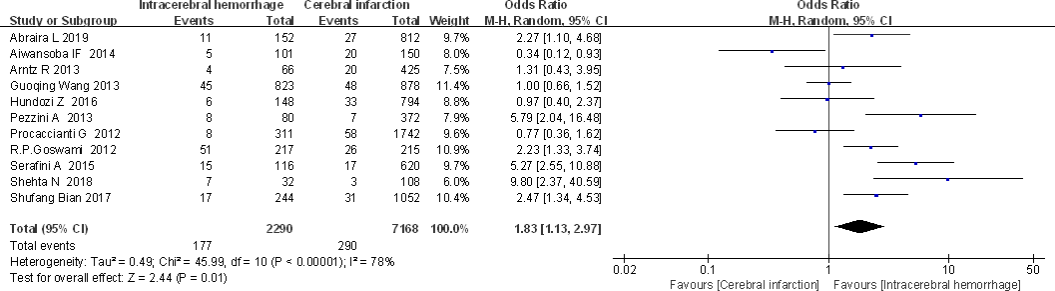
Forest plot: stroke subtype and ES

Eleven (Abraira et al., 2020; Aiwansoba & Chukwuyem, 2014; Arntz et al., 2013; Bian, 2017; Goswami et al., 2012; Hundozi et al., 2016; Pezzini et al., 2013; Procaccianti et al., 2012; Serafini et al., 2015; Shehta et al., 2018; Wang et al., 2013) studies reported stroke subtypes (cerebral hemorrhage/infarction) and the occurrence of ES (Figure 6). In this study, a total of 2,290 cases were included in cerebral hemorrhage group, and 177 were ES patients related to cerebral hemorrhage. A total of 7,168 cases were included in the cerebral infarction group, and 290 of these were ES patients related to cerebral infarction. Heterogeneity test showed significance (*I* ^2^ = 78%, *p* < .000 01), and random‐effects model was used. The results showed that the probability of ES patients in cerebral hemorrhage group was greater than that in the cerebral infarction group [OR (95% Cl) = 1.83 (1.13, 2.97)]. The difference was statistically significant (*p* < .01), indicating cerebral hemorrhage as a risk factor for ES.

**FIGURE 7:**
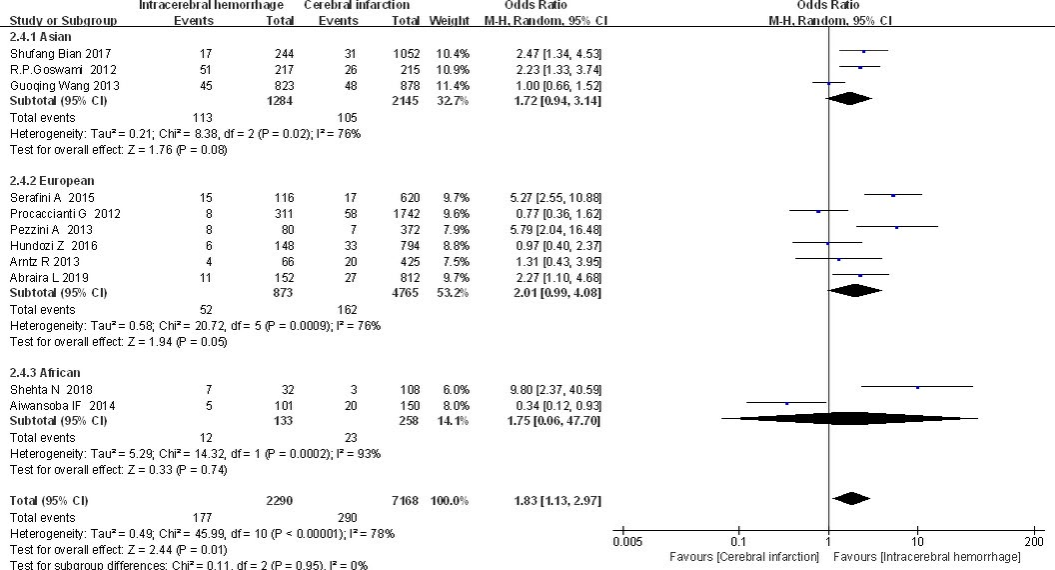
Stroke subtype and ES: Subgroup analysis by region

The heterogeneity of this study (*I* ^2^ = 76%, *p* < .000 01) is obvious, so we further analyze the subgroups according to the total sample size and different regions (Asia/Europe/Africa). The results revealed that the total sample size and the population of different continents have no influence on the outcome indicators. However, comparison of OR between the states through data showed Asia [OR (95% Cl) = 1.72 (0.94, 3.14)], Europe [OR (95% Cl) = 2.01 (0.99, 4.08)], and Africa [OR (95% Cl) = 1.75 (0.06, 47.70)], and it can be found that ES caused by cerebral hemorrhage more likely occurs in the European population, followed by Africa and finally Asia (Figure 7).

#### 3.3.5 Factors related to treatment

Two studies (Bian, 2017; Yang, 2012) have investigated the relationship between receiving surgical treatment and occurrence of ES (Table 6), *I* ^2^ = 30%, *p* = .23, in which no significant heterogeneity was observed, and so fixed‐effects model was used. The results showed no statistical significance [OR (95% Cl) = 1.22 (0.62, 2.41), *p* = .56], suggesting surgical treatment as not risk factor for ES.

**TABLE 6:** Meta‐analysis results: Factors related to treatment

Risk factors	Number of documents involved	Heterogeneity test			Effect model	OR (95%Cl)	*p*
		*Q*	*p*	*I* ^2^ (%)			
Receiving surgical treatment	2	1.42	0.23	30	FE	1.22 (0.62, 2.41)	.56
Reperfusion therapy	2	0.46	0.50	0	FE	1.30 (0.75, 2.55)	.35

Two articles (Abraira et al., 2020; Hundozi et al., 2016) carried out a study on the relationship between patients receiving reperfusion therapy and the occurrence of ES (Table 6), and the results showed that there is no heterogeneity in receiving reperfusion therapy factors (*I* ^2^ = 0, *p* = .50), so the FE model was used for meta‐analysis The results were not statistically significant [OR (95% Cl) = 1.30 (0.75, 2.25), *p* = .35], suggesting that reperfusion therapy has nothing to do with the occurrence of ES.

### 3.4 Sensitivity analysis

For each risk factor, fixed‐effects and random‐effects models were used to calculate the combined OR value and 95% CI (Table 7). The results showed that the OR (95% CI) value of fixed‐effects model of alcoholism was 1.83 (1.30–2.58), which showed statistical significance. While the OR (95%CI) value of random‐effects model was 2.46 (0.46–13.14), which included invalid value 1 and showed no statistical significance. This suggested that the research results of this factor of alcoholism are unstable. The fixed‐effects and random‐effects results of the remaining risk factors are close, indicating that the conclusion of the study was relatively steady.

**TABLE 7:** Sensitivity analysis

Risk factors		Fixed effect model		Random effects model	
		OR	95%Cl	OR	95%Cl
Baseline characteristics	Gender	1.00	0.84–1.20	0.99	0.83–1.19
	Age*	−0.54	−0.27–0.99	−0.54	−0.27–0.99
	NIHSS at admission*	−0.37	−1.29–0.54	1.44	−2.21–5.08
Lifestyle habits	Alcoholism	1.83	1.30–2.58	2.46	0.46–13.14
	Smoking	1.02	0.80–1.31	1.05	0.81–1.36
Basic diseases	Hypertension	0.91	0.74–1.12	0.91	0.74–1.12
	Diabetes	1.23	0.98–1.53	1.18	0.82–1.69
	Atrial fibrillation	0.81	0.57–1.14	0.78	0.46–1.34
	Dyslipidemia	0.92	0.67–1.26	0.92	0.67–1.27
Related to the brain	Cortical injury	4.03	3.26–4.98	5.00	2.85–8.74
	Hemorrhagic transformation	2.77	1.87–4.11	2.92	1.97–4.34
	Stroke subtype	1.56	1.27–1.92	1.83	1.13–2.97
Related to treatment	Receiving surgical treatment	1.22	0.62–2.41	1.41	0.57–3.48
	Reperfusion therapy	1.30	0.75–2.25	1.32	0.77–2.27

Note

The data effect size of the risk factors marked with “*” is MD (95% Cl).

### 3.5 Publication bias analysis

**FIGURE 8:**
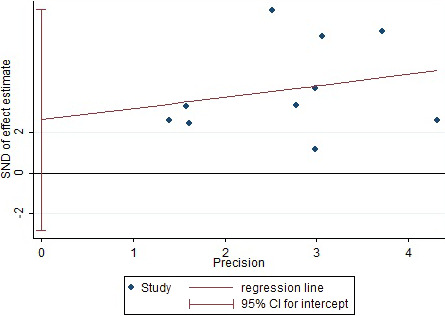
Egger funnel chart (cortical injury)

**FIGURE 9:**
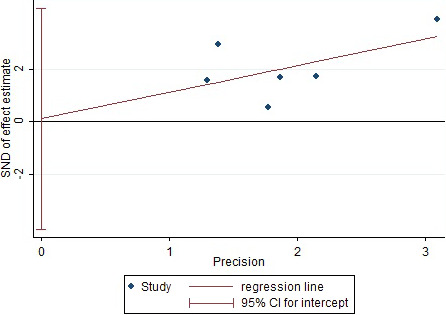
Egger funnel chart (hemorrhagic transformation)

**FIGURE 10:**
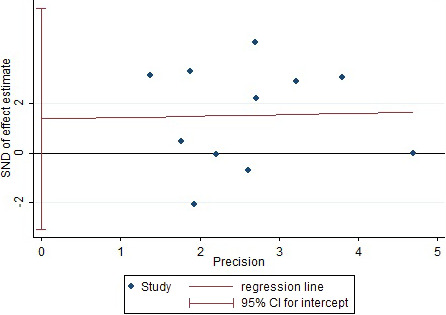
Egger funnel chart (stroke subtype)

The risk factors that included in more than five articles were selected and used Egger method to conduct publication bias test and statistical results of Egger test *p*‐value (Table 8). The three risk factors of cortical injury, cerebral infarction with hemorrhagic transformation were taken, and stroke subtype as example to draw Egger funnel diagram (Figures 8, 9, 10). The Egger test results in Figures 8, 9, and 10 showed that their *p*‐values are equal to 0.300, 0.942, and 0.502, respectively, and no obvious asymmetry in the funnel chart was observed, indicating no obvious publication bias.

**TABLE 8:** Egger test result table

Risk factors	Number of documents involved	*p* value
Gender	13	.694
Age	8	.082
Smoking	9	.183
Hypertension	10	.337
Diabetes	10	.604
Dyslipidemia	6	.911
Cortical injury	10	.300
Hemorrhagic transformation	6	.942
Stroke subtype	11	.502

## 4 DISCUSSION

The pathogenesis of ES after stroke still remained unclear (Zelano, 2020). The risk factors for this symptom have not been fully confirmed. Therefore, what is of great significance for the early identification and targeted prevention and treatment of ES is to carry out the research on the risk factors of this increasingly concerned symptoms of stroke patients.

The pathophysiology of seizures after stroke still remained unclear. If a stroke occurs then there might be various causes of neuronal damage (Reddy et al., 2017), leading to the occurrence of early seizures after stroke. The importance of cortical injury in seizures after stroke has been emphasized (Kwon et al., 2020). The possible mechanism involves increase in glutamate concentration of the extracellular excitatory neurotransmitter, resulting in excitotoxicity of glutamate. Another study found (Kamp et al., 2012) that damage to the stroke cortex in rodent models caused changes in ion channels within a day. If ion channels are damaged, then the concentration of ions present inside and outside the cell's change, leading to depolarization of neurons and promotion of seizures. The researchers such as E Haapaniemi et al. (2014) also confirmed cortical hemorrhage as a risk factor for early seizures. Meta‐analysis results showed that cortical damage as a risk factor for ES. The analysis in various studies, whether cortical damage is caused by hemorrhage or ischemia itself, or cortical damage caused by surgery or external trauma, should also be considered. As there is no clear record of the literature, there is no way to confirm the accuracy and specificity of this idea. Cortical damage is shown to be closely related to the occurrence of ES and acts as a risk factor for ES.

Some studies (Alberti et al., 2008; Bian, 2017; Mohamed & Kissani, 2015) believe that cerebral infarction with hemorrhagic transformation is also the cause of ES. The pathogenesis of ES may be related to the persistent presence of a large amount of glutamate and the release of high levels of excitotoxicity neurotransmitters in the ischemic injury area (Rodríguez Lucci et al., 2018). Procaccianti et al. (2012) believe that cerebral ischemia with hemorrhagic transformation can cause the occurrence of ES. They found that patients with ischemic stroke may be due to the effect of blood degradation products on cortical neurons, which leads to the occurrence of ES. The study included six literatures review their relevance, results showed that with hemorrhagic transformation of cerebral infarction is a risk factor of ES.

The mechanism of cerebral hemorrhage that causes seizures has not been established. Rodríguez Lucci et al. (2018) has believed that ES is caused by destroying the integrity of neurovascular units and cell biochemical dysfunction. The results of two studies conducted by Goswami et al. (2012) and Lekoubou et al. (2020) might explain the increased incidence of ES in patients with cerebral hemorrhage. An earlier study (Berger et al., 1988) suggested that almost all intracerebral hemorrhage associated seizures occurred within a short time after the onset of the intracerebral hemorrhage. Some studies have shown that about 50%–70% of ES will occur in the first 24 hr (Gilmore et al., 2010; Vespa et al., 2003). This meta‐analysis included 11 studies on stroke subtypes (cerebral hemorrhage/cerebral infarction) and the occurrence of ES. The study found cerebral hemorrhage as a risk factor for ES.

Regarding baseline characteristic factors such as age and NIHSS at admission, factors related to smoking and drinking habits, basic disease factors such as hypertension, diabetes, dyslipidemia, and treatment‐related factors, it is proved that the above factors have no obvious relationship with the occurrence of ES in this study. Some studies (Alberti et al., 2008; Denier et al., 2010) have reached similar conclusions on the age factor. Other study (Hundozi et al., 2016) on risk factors for ES when patients are younger than 65 have also been reported. The reason for this situation may be due to the inconsistency between the statistical indicators of the age factor selected in this article, which needs further investigation. Regarding the severity of stroke, this study found that the factor of the NIHSS at admission have no significant relationship with the occurrence of ES, which is consistent with the conclusions of Denier et al. (2010) and Gupta et al. (1988). However, a subgroup analysis based on sample size was carried out and found that there is a correlation between the degree of neurological damage and the occurrence of ES when the sample size more than 500. Arntz et al. (2013) et al. also found that ES was related to the initial severity of stroke. The severity of stroke in ES patients was significantly higher than that of non‐ES patients. In clinical case studies, research to further increase the sample size needs to be carried out in the future. Regarding the underlying disease factors, the study (Beghi et al., 2011) have suggested that hyperlipidemia is a protective factor for hemorrhagic stroke. Studies (Chen et al., 2016; Goswami et al., 2012; Shmuely et al., 2017) have shown that there is a high correlation between hypertension, diabetes, heart disease and the occurrence of ES. Nass et al. (2019) believe that epileptic seizures can cause abnormal changes in blood pressure, which may be caused by epileptic activity that stimulates or inhibits the function of the central nervous system and spreads to different neuronal networks. Therefore, the inconsistency of the conclusions requires further multi‐center and large‐sample research. Regarding reperfusion therapy factor, we conducted a meta‐analysis and found that it has no correlation with the occurrence of ES. In this study, only two articles that meet the requirements were included. Therefore, more prospective studies are needed to confirm whether reperfusion therapy is a risk factor for ES for early identification or prediction.

With regard to alcoholism, due to inconsistent results of fixed‐effects and random‐effects calculations, the results of meta‐analysis remained unstable. This is because too few study samples were included, or the definition of alcoholism is still inconsistent or not described. Researcher Zhang et al. (2014) have found alcoholism (OR = 1.70, 95% CI = 1.23–2.34, *p* < .01) as a risk factor for ES after stroke through systematic review and meta‐analysis. But our study showed no statistical significance between alcoholism and ES. Therefore, whether alcoholism is related to the occurrence of ES, whether it acts as a risk factor for ES and the underlying mechanism of alcoholism that leads to seizures requires further research.

### 4.1 Limitations

There are still many deficiencies that should be acknowledged in this study and are as follows: (1) all the included studies are retrospective case‐control studies; (2) in several studies, reports have shown that seizures were recorded by family or caregivers during the follow‐up period; (3) some risk factors involving less literature, such as four articles on alcoholism and atrial fibrillation and two on surgical treatment, might lead to a certain bias in the meta‐analysis; (4) regarding the definition of ES, most of the studies included e seizures that occurred within 7 days after stroke onset according to the definition of the International League Against Epilepsy, but a few studies did not follow the definition of the International League Against Epilepsy, leading to some bias; and (5) the studies included 11 countries with three continents, with a large geographical span, large differences in climate and human environment, and might not be representative of a single country. Therefore, future multi‐center, large‐sample epidemiological studies are needed to further clarify the risk factors for ES after stroke.

## 5 CONCLUSION

In summary, the results of this meta‐analysis indicate that cortical injury, cerebral infarction with hemorrhagic transformation, and cerebral hemorrhage are closely related to the occurrence of ES, and are risk factors and important predictors for the occurrence of ES. Medical staff can refer to the results of this study to identify early, targeted risk groups of early‐onset seizures after stroke, reduce early incidence of seizures in patients after stroke and thus improve the quality of life of patients and their families.

## CONFLICT OF INTEREST

The authors declare that they have no competing interests.

## AUTHORS' CONTRIBUTIONS

FAN Xiaoxuan and MA Sitian contributed to the conception of the study; MA Sitian 、WANG Huan 、YANG Yongfeng and WANG Kai performed the selection of documents and the evaluation of document quality; FAN Xiaoxuan and MA Sitian contributed significantly to analysis and manuscript preparation; MA Sitian performed the data analyses and wrote the manuscript; ZHAO Xiaoping and FAN Xiaoxuan helped perform the analysis with constructive discussions.

## ETHICAL APPROVAL

Not applicable.

## CONSENT FOR PUBLICATION

Not applicable.

### PEER REVIEW

The peer review history for this article is available at https://publons.com/publon/10.1002/brb3.2142.

## Data Availability

All data generated or analyzed during this study are included in this published article and its supplementary information files.
